# Network-centered homeostasis through inhibition maintains hippocampal spatial map and cortical circuit function

**DOI:** 10.1016/j.celrep.2021.109577

**Published:** 2021-08-24

**Authors:** Klara Kaleb, Victor Pedrosa, Claudia Clopath

**Affiliations:** 1 Bioengineering Department, Imperial College London, London, UK; 2 Sainsbury Wellcome Centre, UCL, London, UK

**Keywords:** network homeostasis, inhibitory plasticity, hippocampus, place cells, remapping, recurrent networks

## Abstract

Despite ongoing experiential change, neural activity maintains remarkable stability. Although this is thought to be mediated by homeostatic plasticity, what aspect of neural activity is conserved and how the flexibility necessary for learning and memory is maintained is not fully understood. Experimental studies suggest that there exists network-centered, in addition to the well-studied neuron-centered, control. Here we computationally study such a potential mechanism: input-dependent inhibitory plasticity (IDIP). In a hippocampal model, we show that IDIP can explain the emergence of active and silent place cells as well as remapping following silencing of active place cells. Furthermore, we show that IDIP can also stabilize recurrent dynamics while preserving firing rate heterogeneity and stimulus representation, as well as persistent activity after memory encoding. Hence, the establishment of global network balance with IDIP has diverse functional implications and may be able to explain experimental phenomena across different brain areas.

## Highlights

• Input-dependent inhibitory plasticity (IDIP) provides network-wide homeostasis
• IDIP can explain hippocampal remapping following place map silencing
• IDIP can also provide recurrent network homeostasis with firing rate diversity

## Graphical abstract

**Figure:**
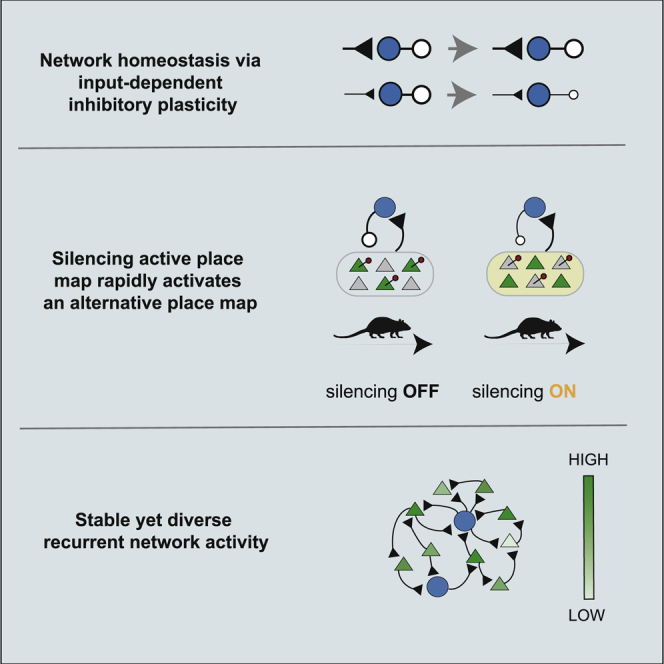

## Teaser

Kaleb et al. show that the input-dependent inhibitory plasticity model can explain the network-centered homeostasis of firing rates reported upon optogenetic silencing of hippocampal place maps. They extend the same model to recurrent cortical networks and make experimentally testable predictions.

## Introduction

Although neural activity varies, it is usually limited to a small operational range of a few hertz. Deviations from this range are often associated with pathological states (Wondolowski and Dickman, 2013). Given that neural activity undergoes constant experiential change, there is a requirement for active processes to maintain stability. Many such processes have been identified, such as synaptic scaling (Turrigiano et al., 1998; Desai et al., 2002; Turrigiano and Nelson, 2004; Goel and Lee, 2007; Glazewski et al., 2017), intrinsic plasticity (Desai et al., 1999; Gainey et al., 2018; Lambo and Turrigiano, 2013; Maffei and Turrigiano, 2008), meta-plasticity (Bienenstock et al., 1982; Kirkwood et al., 1996; Zenke et al., 2013; Frank et al., 2006), diffusive neuromodulation (Sweeney et al., 2015; Steinert et al., 2008, 2011; Naumann and Sprekeler 2020), structural plasticity (Yin and Yuan 2015; Gallinaro and Rotter 2018), and inhibitory plasticity (Woodin et al., 2003; Maffei et al., 2004, 2006; Chen et al., 2011; Vogels et al., 2011; Keck et al., 2011; van Versendaal et al., 2012; D’amour and Froemke 2015; Udakis et al., 2020; Clopath et al., 2016; Hennequin et al., 2017; Das et al., 2011; Haas et al., 2006). All of these together make up the term homeostatic plasticity and, although vastly different, they all act as a negative feedback mechanism that adjusts the neural parameters to compensate for deviations from some set point. Homeostatic plasticity is often studied in the context of neuron-centered control because individual neurons return to their preferred level of activity after experimental manipulation (Hengen et al., 2013; Pacheco et al., 2021). There is also experimental evidence of network-centered homeostasis (Hirase et al., 2001; Slomowitz et al., 2015; Trouche et al., 2016), where the mean activity of the whole network is homeostatically maintained. However, computational studies of such mechanisms are few (Sweeney et al., 2015; Mackwood, 2019; Naumann and Sprekeler, 2020); thus, they remain less well understood.

To illustrate network-centered homeostasis, we turn to the hippocampus. Spatial environments are known to be represented by a cognitive map consisting of a subset of hippocampal pyramidal cells. These are called place cells, as they fire action potentials when the animal is in a specific location within the environment, their place fields, and, thus form a place map (O’Keefe and Dostrovsky, 1971; O’Keefe, 1976; O’keefe and Nadel, 1978; Wilson and McNaughton, 1993). Optogenetic silencing of the CA1 place cells encoding a familiar environment leads to rapid activation of previously silent cells, followed by a slower, seconds-long activity change toward a stable level on par with that of the original place map (Trouche et al., 2016). Thus, an alternative place map transiently emerges while the original place cells are being silenced, and the spatial representation is homeostatically maintained. Furthermore, with repeated silencing, the alternative place map is consolidated over the original place map (Trouche et al., 2016). Because neurons presumably have access only to their own activity, it is unclear how such a perturbation can be detected and compensated for at the network level.

Likely candidates to implement such network-centered homeostasis are inhibitory neurons. CA1 inhibitory neurons are strongly connected to a large number of heterogeneously tuned CA1 pyramidal cells (Ali et al., 1998; Freund and Buzsáki, 1996; Gulyás et al., 1999; Bezaire and Soltesz, 2013; Csicsvari et al., 1998; English et al., 2017) and exhibit broad spatial tuning (Grienberger et al., 2017). Thus, they can sense and influence the activity of their local network. Neuron-centered inhibitory plasticity has been shown to provide highly efficient homeostasis (Vogels et al., 2011). However, there are indications that inhibitory plasticity may also act more globally. For instance, hippocampal disinhibition has been reported when global but not single-neuron activity is suppressed (Hartman et al., 2006; Peng et al., 2010). Moreover, in the highly recurrent networks of the neocortex, where inhibitory neurons also feature strong connectivity (Sohya et al., 2007; Niell and Stryker, 2008; Kerlin et al., 2010; Ma et al., 2010; Zariwala et al., 2011; Znamenskiy et al., 2018; Wilson et al., 2017; Fino and Yuste, 2011; Packer and Yuste, 2011; Hofer et al., 2011; Bock et al., 2011; Pfeffer et al., 2013), it has been shown that inhibitory scaling may be decoupled from unique postsynaptic neuron activity (Joseph and Turrigiano 2017). Lastly, sensory deprivation studies across the primary cortices (Kuhlman et al., 2013; Barnes et al., 2015; Li et al., 2014; Gainey et al., 2018) have led to the suggestion that inhibition could be broadly adjusted as a function of network activity (Gainey and Feldman, 2017). Inspired by these experimental findings, we hypothesize that the synaptic input to the inhibitory neurons could act as a proxy for the local network activity and, therefore, be used to appropriately adjust the level of inhibition. Such plasticity could occur through depolarization-induced modulation of spike-evoked inhibitory transmission (Christie et al., 2011; Bouhours et al., 2011; Rowan and Christie, 2017) or inhibitory neuron intrinsic excitability (Gainey et al., 2018).

In this work, we computationally study the properties and potential functionality of such plasticity, which we term input-dependent inhibitory plasticity (IDIP). We show that IDIP provides a mechanistic explanation for the emergence of active and silent place cells in a hippocampal CA1 network model. Our model also reproduces the fast and reversible remapping following acute optogenetic place map silencing, as well as the alternative place map consolidation following repeated silencing (Trouche et al., 2016). Furthermore, we show that IDIP in a cortical recurrent network model provides rapid firing rate homeostasis while maintaining important network features, such as firing rate heterogeneity and persistent activity. Thus, we show that IDIP allows for accurate maintenance of neural representation while preserving the flexibility important for neural coding.

## Results

### The IDIP rule as a homeostatic mechanism

**Figure 1:**
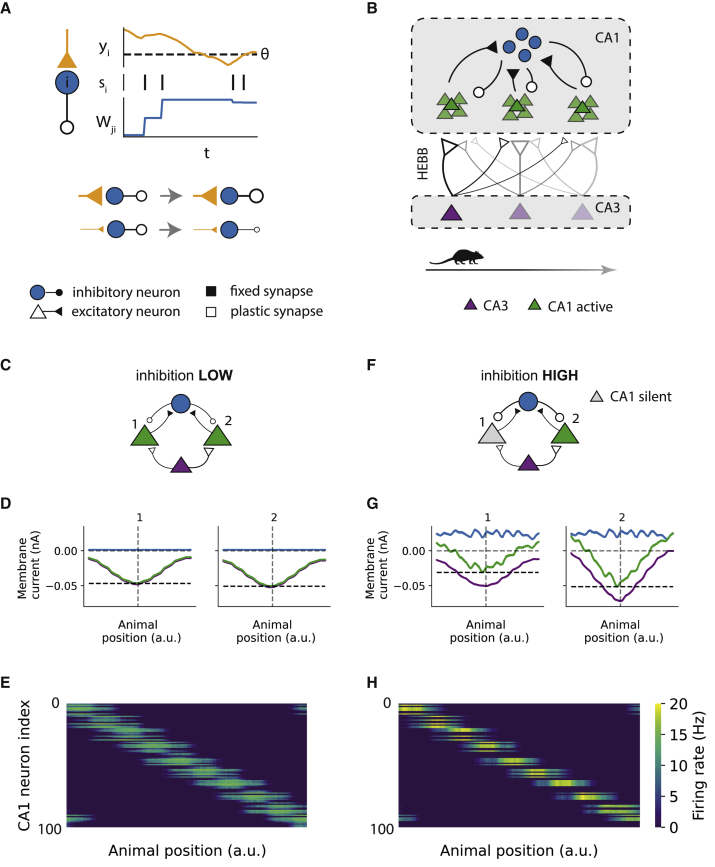
The IDIP rule enables emergence of active and silent place cells in a hippocampal network (A) Inhibitory plasticity rule diagram. At every spike (s), an inhibitory neuron i(blue) adjusts its inhibitory synaptic weights based on the synaptic input it receives (y) and its target input θ. (B) Hippocampal network diagram. CA3 excitatory neurons (purple) receive place-dependent external current. CA3 neurons project to uniquely tuned CA1 excitatory neurons (green), which are interconnected with CA1 inhibitory neurons (blue). The CA3-to-CA1 excitatory synapses are plastic under Hebbian plasticity, and the CA1 inhibitory synapses are plastic under IDIP. (C–E) State of the network during the first lap, before any learning. (C) A diagram of a sample microcircuit found in the network in (B), consisting of 2 CA1 place cells (1 and 2) with the same spatial tuning but with cell 2 having stronger tuning than cell 1. (D) The excitatory (purple), inhibitory (blue), and net (green) currents received by cells 1 and 2 during the first lap on the track. The higher peak net current (black dashed line) received by cell 2 reflects its stronger tuning. (E) The firing rates of all CA1 place cells (y axis) during the first lap (x axis). All place cells are active but with varying amplitudes, reflecting differences in their place tuning strengths. (F–H) The same as in (C)–(E) but after learning. Because of increased inhibition, silent place cells (gray) emerge as the difference in the peak net currents from the first lap (D) is amplified with learning.

Experimental studies suggest the existence of network-centered homeostasis (Hirase et al., 2001; Slomowitz et al., 2015; Trouche et al., 2016), where the mean firing rate of the network, rather than that of individual neurons, is homeostatically maintained. For example, rapid homeostasis of place representation in the hippocampal CA1 region is observed after optogenetic silencing of a familiar place map, through emergence of an alternative place map (Trouche et al., 2016). Likely candidates underlying such a mechanism are inhibitory neurons, because of their dense inter-connectivity with the surrounding pyramidal cells. Such global regulation of network activity could be achieved through modulation of inhibition as a function of the synaptic input the inhibitory neurons receive. We hypothesize an inhibitory plasticity mechanism where strong inputs onto inhibitory neurons lead to strengthening of the inhibitory output, whereas weak inputs onto the inhibitory neurons lead to weakening of the inhibitory output. For simplicity, we choose to implement this IDIP rule by scaling the inhibitory synaptic weights as a function of the difference between the synaptic input the inhibitory neuron receives and a set target input value (Figure 1A).

### The IDIP rule allows for emergence of active and silent place cells in a hippocampal network

To assess whether IDIP can lead to emergence of active and silent place cells, we build a hippocampal network model of leaky integrate-and-fire neurons. The network model consists of the CA3 and CA1 regions (Figure 1B). Each pyramidal cell in the CA3 region receives unique place-tuned external current (Figures S1A and S1B), representing the location of a simulated mouse on a 1D annular track with equally spaced place fields. The CA3 neurons project to the CA1 excitatory neurons, which are divided into equally sized groups, with neurons in each group tuned to the same place field. However, our simple model does not capture all of the heterogeneity of tuning present in a biological system. As the CA1 excitatory neurons are poorly recurrently connected (Deuchars and Thomson, 1996; Thomson and Radpour, 1991), we assume that there are no recurrent connections between them. The CA1 excitatory neurons project to the CA1 inhibitory neurons and vice versa. The CA1 inhibitory neurons have no spatial tuning (Figure S1K), in agreement with previous experiments (Dupret et al., 2013; Grienberger et al., 2017). After the first lap on the track, the excitatory synapses between the CA3 and CA1 excitatory neurons and the inhibitory synapses to the CA1 excitatory neurons are made plastic with Hebbian plasticity and IDIP, respectively.

We first tested whether IDIP could allow for the emergence of active and silent CA1 place cells. The experiments suggest that place cells that later become active or silent are differentiable even before the first exploration of the environment (Epsztein et al., 2011). We incorporate this in our model by introducing variability in the amplitude of CA1 place tuning (Figures 1C, 1D, S1C, and S1D). Because the initial inhibitory weights are set to low values, all place cells are active in the first lap on the track (Figure 1E). Thus, the synaptic input received by the CA1 inhibitory neurons is high and above the target input (Figure S1J). We then turn on the Hebbian plasticity and IDIP and simulate 100 laps on the track, which we term the exploration phase. During this phase, the place cells increase their tuning and activity in a positive feedback loop characteristic of Hebbian learning (Figures 1F, 1G, and S1E–S1H). This further increases the input to the CA1 inhibitory neurons and, hence, increases the inhibitory synaptic weights through IDIP to maintain the target input (Figures S1I and S1J). Because place cells with initially weaker place tuning are less active, they are unable to escape the increasing lateral inhibition. Thus, stable active and silent place cells form within each CA1 place-tuned group (Figure 1H). The number of active place cells is a function of the target input to the CA1 inhibitory neurons (Figure S1L). Hence, we show that IDIP, together with Hebbian plasticity and place-tuned inputs, can explain the formation of active and silent place cells in a hippocampal network model.

### The IDIP rule enables rapid homeostatic remapping during active place cell silencing

**Figure 2:**
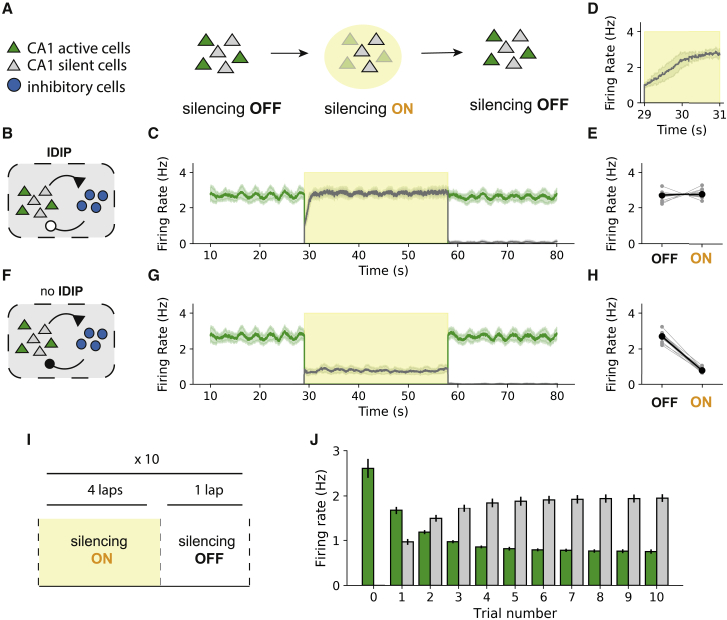
Active place cells silencing leads to rapid emergence of an alternative place map (A) Silencing protocol diagram. All active cells (green) are silenced for one lap (yellow) and released in the subsequent lap. (B–E) Silencing in the network following IDIP. (B) Network diagram after the exploration phase (100 laps). (C) Mean firing rate of the active (green) and silent (gray) place maps ± STD over 20 networks just before, during (yellow ), and after silencing. (D) Alternative place map activation just after silencing. (E) The mean firing rate of the place map before (OFF) and during (ON) silencing. Gray circles indicate individual networks. Black circles indicate the average over 20 networks. (F, G, H) Same as in (B), (C), and (E) but without IDIP during silencing. (I) Consolidation protocol diagram. The established place map is silenced for 4 consecutive laps and then released for a single testing lap. This is repeated for 10 trials. (J) Mean firing rate of active (green) and silent (gray) place cells during the testing laps at each trial. Trial 0 corresponds to the activity of the network just after the exploration phase. The error bars denote ± STD over 20 networks.

To assess whether the proposed IDIP rule can facilitate rapid place cell remapping, we silence all active place cells after the exploration phase (Figure 2A), as in Trouche et al. (2016). An alternative map emerges in networks with (Figures 2B and 2C) and without (Figures 2F and 2G) IDIP. However, the degree of its activation depends on inhibitory plasticity. In the networks with IDIP during silencing (Figures 2B–2E), the dynamics of alternative place map activation are similar to those observed experimentally (Figure 2C; Trouche et al., 2016). The firing rates of the alternative place cells first increase almost immediately after silencing onset (fast phase) and increase further within the next 1–2 s (slow phase) (Figures 2C and 2D). In our model, the fast phase is due to the rapid decrease in the CA1 inhibitory neurons firing rate (Figure S2A) because the originally active place cells that were driving their activity are no longer active. Thus the alternative place map emerges, but at a lower firing rate than the original place map because the silent place cells are less sharply place tuned than the active place cells. In networks without IDIP during silencing (Figures 2F–2H), the activity of the alternative place map does not progress beyond this phase (Figures 2G and 2H). However, with IDIP, a second, slower phase occurs (Figures 2C and 2D) as the level of inhibition is adjusted to the lower input from the alternative place map (Figure S2B). Therefore, the final activity of the alternative place map matches that of the original place map (Figure 2E). Hence, IDIP acts as a homeostatic mechanism to maintain network activity during acute silencing.

When we turn off silencing, the original place map re-emerges (Figure 2C), in agreement with experimental data (Trouche et al., 2016). However, repeating the silencing protocol (Figure 2I) consolidates the alternative place map (Figure 2J), also in agreement with experimental data (Trouche et al., 2016). In our model, this happens through the gradual activity-dependent Hebbian plasticity in the CA3-to-CA1 excitatory synapses (Figures S2D and S2E). Therefore, our model suggests that synaptic plasticity of feedforward inputs onto CA1 place cells is a good candidate for the mechanism underlying place map consolidation.

### The IDIP rule establishes global E/I balance in recurrent networks

**Figure 3:**
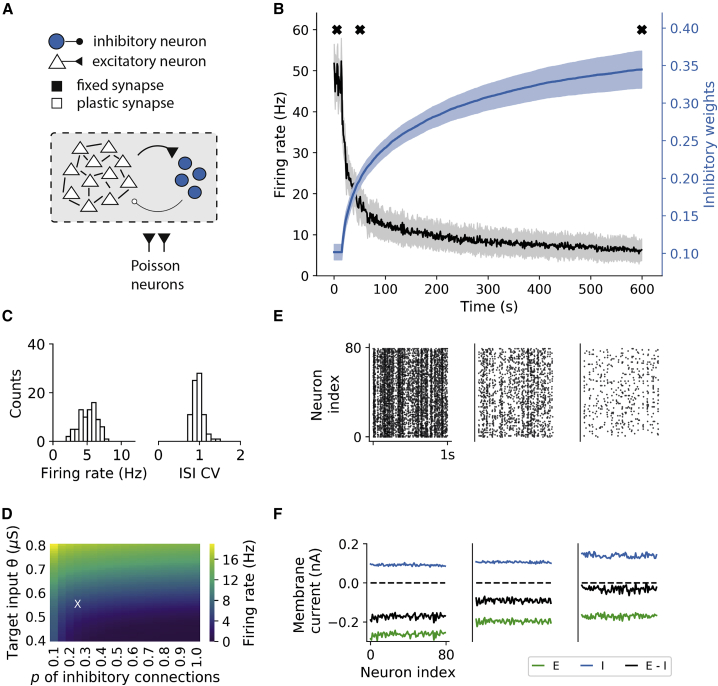
The IDIP rule establishes global E/I balance in recurrent networks (A) A recurrent network diagram with excitatory (white) and inhibitory (blue) neurons receiving external input from a pool of Poisson excitatory neurons. Only the inhibitory synaptic weights are made plastic with IDIP. (B) The evolution of the mean excitatory firing rate (black) and the mean inhibitory weight (blue) of the network ± STD across all units. IDIP is turned on at 15 s. (C) After learning, the excitatory firing rate stabilizes to a mean value of 5.2 Hz (left). The network displays an asynchronous firing pattern (right). (D) The mean firing rate across 20 networks with varying input target value (y axis) and inhibitory connectivity (x axis). X marks the parameter combination used in our simulations. (E) Spike raster plots at the three time points (X) marked in (B). (F) Mean membrane currents as a function of excitatory neuron index at three the time points (X) marked in (B).

As there is also some evidence of network-centered control of cortical activity through inhibition (Joseph and Turrigiano, 2017; Gainey and Feldman, 2017; Gainey et al., 2018), we wanted to assess whether the proposed IDIP learning rule can homeostatically regulate such circuits. To this end, we simulate a sparsely connected recurrent network (Figure 3A). Each neuron in the network receives large external excitatory input, and the initial inhibitory synaptic weights are set to low values. Thus, without any plasticity, the network exhibits pathologically high activity (Figure 3B). As the inhibitory neurons consequently receive very large excitatory synaptic input, IDIP increases the inhibitory synaptic weights (Figures 3B and S3A). The network, therefore, progresses from high synchronous to low asynchronous firing (Figure 3E). The excitatory firing rates reach a more physiological regimen with a reasonable firing rate distribution and irregular firing rate dynamics (Figures 3B and 3C). Inhibitory neurons in the network also exhibit a diversity of firing rates and irregular firing rate dynamics (Figures S3B and S3C). Adding inhibitory-inhibitory connections to the network, increasing the network size, or changing the learning rate does not change our results (Figures S3E, S3F, and S3I). Hence, IDIP can homeostatically regulate network-wide activity in recurrent networks.

The proposed IDIP rule does not impose a unique target firing rate for each neuron in the network. Instead, it controls the mean firing rate across the whole network. The final variability in the net current received by each individual excitatory neuron after inhibitory learning (Figure 3F) results in firing rate diversity (Figure 3C). This is in contrast with networks following a neuron-centered rule, such as inhibitory spike-timing-dependent plasticity (iSTDP) (Vogels et al., 2011; Figures S3E, and S3G). To assess the range of the firing rates IDIP can support, we simulate the network with various values of inhibitory target input. Higher values of inhibitory target input lead to higher network activity (Figure 3D, y axis). Hence, the activity of the network following IDIP depends on the target input of the inhibitory neurons. Moreover, higher inhibitory connectivity decreases the final mean and standard deviation of network activity (Figures 3D and S3D, x axis), whereas excitatory connectivity has no effect (Figure S3H). Thus, IDIP establishes a global rather than detailed network excitatory (E)/inhibitory (I) balance while allowing for network diversity.

### The IDIP rule enables maintenance of neural representations

**Figure 4:**
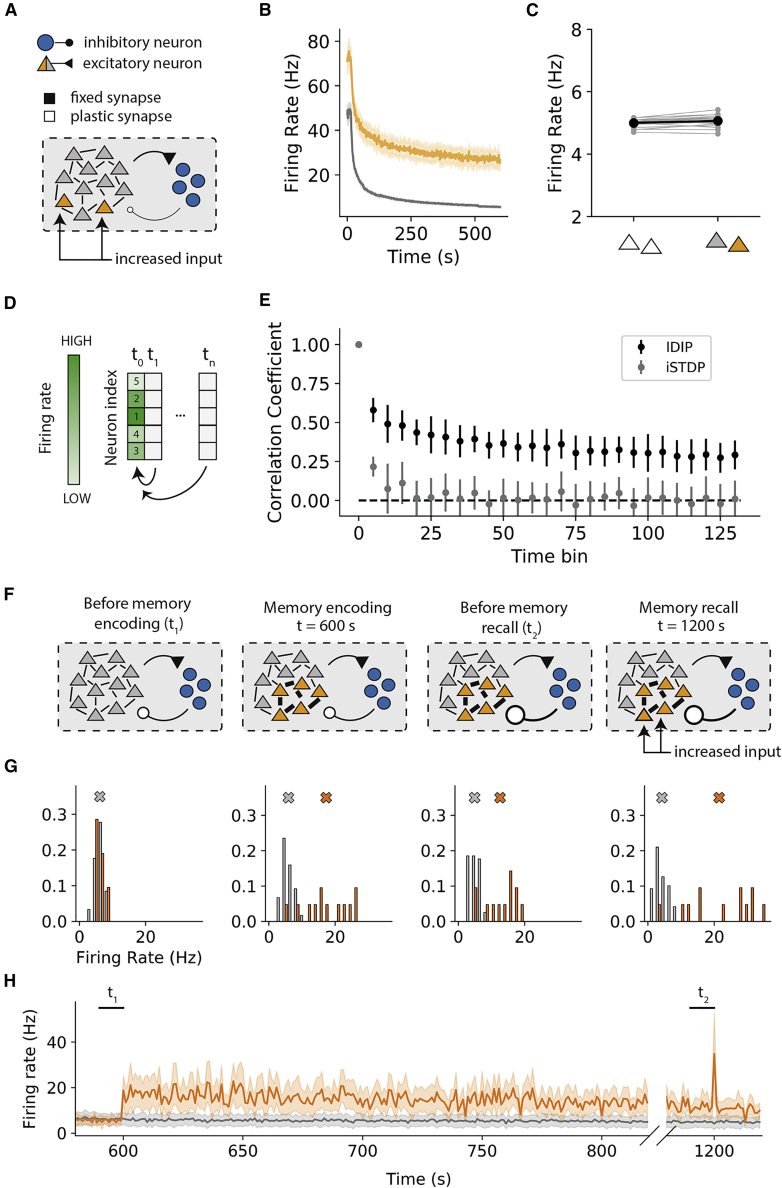
The IDIP rule enables maintenance of neural representations and memory trace persistence (A) The same network as in Figure 3A but with a subset of excitatory neurons (orange) receiving increased external input. (B) The mean firing rates of the two network subsets, color coded as in (A). Bold lines denote the mean, and the shaded area indicates ± STD over 20 networks. (C) The mean firing rate of the networks without (left) and with (right) increased input to a subset of neurons. Gray circles indicate individual networks, and black circles indicate the average over 20 networks. (D) Network representation task diagram. We calculate the preservation of the correlation with the initial firing rate rank during the entire course of the simulation. (E) The Spearman rank correlation coefficient for the network following IDIP (black) and iSTDP (gray). Error bars correspond to ± STD over 20 networks. (F–H) Performance of IDIP in an associative memory task. (F) Associative memory task protocol. Following network stabilization, we increase recurrent excitatory connections between a subset of neurons. The encoded memory is then recalled by increasing external input to a subset of neurons within it. (G) Histograms of mean firing rates of the memory ensemble (orange) and the rest of the network (gray) at the four time points in (F). The color-coded crosses indicate the mean firing rates of each subset of neurons. (H) The evolution of the firing rates of the memory ensemble (orange) and the rest of the network (gray) during the memory task. The shaded area indicates ± STD over all neural subset units.

We wanted to assess whether the neural representation could be maintained in the networks following IDIP. To this end, we increase the external drive to a subset of neurons in the network (Figure 4A), which sustain a higher firing rate relative to the rest of the network following inhibitory learning (Figure 4B). This effect is mediated by the highly active neural subset monopolizing the input to inhibitory neurons, leading to greater inhibition to the rest of the network. Thus, the mean network firing rate is maintained (Figure 4C). As expected, the deviations from the target excitatory neuron firing rate are suppressed in the network following iSTDP (Vogels et al., 2011; Figure S4A). Hence, IDIP can control the recurrent network activity while preserving activity heterogeneity.

To assess whether such representation can be conserved across the whole neural population, we rank the firing rates of the excitatory neurons at the beginning of our simulations, before any inhibitory plasticity, and use Spearman’s rank correlation coefficient to measure whether this rank is maintained over time following inhibitory plasticity (Figure 4D). In the networks following IDIP, the rank correlation is largely conserved after inhibitory learning (Figures 4E and S4B). However, in the networks following iSTDP (Vogels et al., 2011), the rank correlation is mostly lost because all neurons converge to similar firing rates (Figures 4E and S4B). Hence, IDIP can preserve heterogeneity in firing rates at the single-neuron level and across the whole network.

### The IDIP rule enables memory trace persistence and recall

We also assess the performance of the recurrent network following IDIP in a simple associative memory task. To this end, we initialize the network as before, but after inhibitory learning, we encode a memory in our network by increasing the recurrent excitatory connections within a subset of neurons (Figure 4F). We then attempt to recall the memory by increasing external input to a subset of the memory ensemble. After encoding, the memory ensemble exhibits sustained activity that is higher than the rest of the network, even after IDIP has converged (Figures 4G and 4H). This is in contrast to the networks following iSTDP (Vogels et al., 2011), in which the activity of the memory ensemble becomes indistinguishable from the rest of the network at convergence (Figures S4D and S4E). Such persistent activity of the memory ensemble has been reported in some experiments (Yassin et al., 2010; Ghandour et al., 2019; but see Barron et al., 2017).

We then test whether we can recall the memory, given that IDIP made inhibition stronger in the network. We show that the memory can be recalled by increasing the external input to a subset of the memory ensemble (Figures 4G and 4H). Performing the same protocol in a network following iSTDP (Vogels et al., 2011) shows that fewer memory cells are re-activated with recall (Figure S4D). Thus, recall in the network following IDIP has higher fidelity. This is in line with previous experiments (Pignatelli et al., 2019), where increased activity of memory cells facilitates greater pattern completion. Hence, IDIP allows sustained activity of memory cells after encoding as well as faithful memory recall from a partial cue.

We show that our proposed inhibitory plasticity rule, IDIP, can homeostatically regulate activity in models of hippocampal and cortical networks. Importantly, using IDIP, we are able to reproduce experimental findings in the hippocampus (Trouche et al., 2016) and propose its potential functional implications in the recurrent cortical networks.

## Discussion

Although the study of neural homeostasis is frequently neuron centered, there is some evidence that network-centered mechanisms are also at play (Hirase et al., 2001; Slomowitz et al., 2015; Trouche et al., 2016). These may be implemented via densely connected inhibitory neurons. In this work, we take inspiration from the experimental data and hypothesize that network homeostasis could be achieved through IDIP, in which inhibition is adjusted as a function of the synaptic input the inhibitory neurons receive. We show that, in a hippocampal CA1 network model, IDIP can provide a mechanistic circuit understanding and reproduce experimental data of active place cell silencing (Trouche et al., 2016). Furthermore, we show that IDIP can also regulate the activity of recurrent neural networks while preserving the flexibility important for neural coding. Altogether, our results suggest that network homeostasis following external manipulation or endogenous changes could share a common underlying mechanism.

In contrast to neuron-centered inhibitory plasticity (Vogels et al., 2011), IDIP features an absence of a target firing rate for each excitatory neuron. The importance of this is seen in our data-driven model of the hippocampal CA1 network. Here, active and silent place cells emerge as neurons with higher activity dominate the input to inhibitory neurons and, thus, recruitment of lateral inhibition. Such competition for recruitment of inhibition has been suggested previously to shape hippocampal assemblies (Buzsáki 2010). Furthermore, silencing of an established place map induces rapid compensatory adjustment of inhibition and, thus, emergence of an alternative place map (Figure 2C), as reported experimentally (Trouche et al., 2016). We show that non-plastic inhibition is not consistent with the experimental data (Figure 2G). Finally, we reproduce the alternative place map consolidation with repeated silencing (Trouche et al., 2016; Figure 2J). In our model, this occurs through gradual changes in the CA3-to-CA1 excitatory synaptic weights (Figures S2D and S2E), facilitated by disinhibition during each silencing lap (Figures S2A and S2B). Thus, over time, the original place map is destabilized, which has been suggested previously as a necessary condition for remapping (Schoenenberger et al., 2016). Hence, network homeostasis through IDIP is a possible explanation for the experimental phenomena in the hippocampus.

Furthermore, we show that IDIP can also regulate the dynamics of the cortical recurrent networks while preserving diversity in network firing rates (Figure 3C). The resulting firing rate distribution is consistent with the broad and heavy-tailed distribution of firing rates observed experimentally (Wohrer et al., 2013). Such a range of activity is thought to be optimal for information storage in the brain (Laughlin, 1981) and enables linear network responses over a broad range of inputs (Sweeney et al., 2015). Due to the absence of a unique firing rate set point, when we increase external inputs to some neurons, they remain consistently more active than the rest of the network (Figure 4B). We show that IDIP can also extend this stability of the firing rate rank across the whole neural population, unlike iSTDP (Vogels et al., 2011; Figure 4E). Importantly, the neural activity diversity is not imposed but emerges because of random network structure. Hence, IDIP provides global control of recurrent network dynamics without a firing rate set point for each neuron, which allows flexibility and, thus, conservation of network representation.

As highlighted throughout this work, dense connectivity of inhibitory neurons to their local networks makes them an ideal candidate for network homeostasis. However, more sparsely connected networks would lead inhibitory neurons to sense a subset of the network and potentially affect a different subset. Thus, the final network firing rate would be less constrained (Figures 3D and S3D). This suggests that the network activity is differentially modulated by IDIP depending on the network architecture. In the limit of very sparse networks, IDIP may not even act as a homeostatic mechanism (Figures S3J–S3L). This may be relevant for prevention of redundancy or for contrast enhancement (Hartline and Ratliff, 1957). Furthermore, inhibitory connectivity is known to vary within (Meyer et al., 2011) and between (Tamamaki and Tomioka, 2010) brain areas. Thus, the IDIP rule would have different functional consequences in different brain regions.

The type of inhibitory plasticity we propose here could be implemented in several different ways. Synaptic input integration over the timescale used in our model could be mediated through N-methyl-D-aspartate receptors (NMDARs), which have been shown to be preferentially localized at feedback synaptic inputs to hippocampal Parvalbumin-positive (PV+) cells (Le Roux et al., 2013) and disrupt spatial representation when knocked out (Korotkova et al., 2010). Moreover, hippocampal PV+ cell NMDARs have been shown to facilitate supralinear dendritic integration from clustered synapses (Cornford et al., 2019). The synaptic plasticity modeled here could occur through a mechanism such as analog-digital facilitation (ADF) (Alle and Geiger, 2006), where the spike-evoked transmission is graded by the presynaptic voltage fluctuations. Indeed, ADF has been reported in inhibitory neurons, where it acts through basal Ca^2+^ accumulation and leads to an increase in synaptic release (Christie et al., 2011; Bouhours et al., 2011) as well as Kv channel inactivation (Rowan and Christie, 2017). Alternatively, IDIP could also act through the plasticity of inhibitory neuron intrinsic excitability. In Figures S2C and S4C, we show that our results are qualitatively robust to variations of our learning rule as long as the plasticity is a function of the inhibitory neuron synaptic input, or its derivative. Thus, in this work, we show the functional outcome of a class of models of IDIP.

Our model makes the following experimental predictions. First, we predict that, during optogenetic silencing of the place map (Trouche et al., 2016), the alternative place map emerges as the level of inhibition decreases. If inhibitory plasticity is blocked during silencing, then we predict that the alternative place map may still emerge but that its activity would be much lower than that of the original place map, as shown in Figures 2G and 2H. Second, we predict that the inhibitory plasticity at play is a function of the synaptic input to the inhibitory neurons. This could be experimentally verified by optogenetically stimulating their presynaptic excitatory cells. Here we predict to see a progressive increase in inhibition to the excitatory cells and vice versa. This may be observed as change in the magnitude of the inhibitory postsynaptic potentials (IPSPs) or inhibitory mean spike threshold. Third, we predict that homeostatic recovery should not disrupt pre-existing network structure, as shown in Figure 4E. Interestingly, this has been reported in a re-analysis of the experimental data from Hengen et al. (2016) by Wu et al. (2020), but it was attributed to synaptic scaling.

Finally, other distinct solutions have been proposed for network-centered homeostasis (Sweeney et al., 2015; Naumann and Sprekeler, 2020). It is very likely that they all coexist alongside other, neuron-centered homeostatic mechanisms and that each mechanism may control distinct aspects of neural function, as shown in a recent computational study (Wu et al., 2020). Hence, it would be of future interest to study IDIP in conjunction with other homeostatic plasticity rules, as their interaction may increase the compensatory repertoire of the networks and endow them with non-trivial emergent properties.

## STAR★Methods

### Key resources table

**Table:** 

REAGENT or RESOURCE	SOURCE	IDENTIFIER
**Software and algorithms**		

Original code	This paper	https://doi.org/10.5281/zenodo.5110063
Python, version 3.6.9	https://www.python.org/	N/A
Numpy, version 1.19.2	https://numpy.org/	N/A

### Resource availability

#### Lead contact

Further information and requests for resources should be directed to and will be fulfilled by the Lead Contact, Claudia Clopath (c.clopath@imperial.ac.uk).

#### Materials availability

This study did not generate new materials.

### Method details

#### Neuron Model

We use the single compartment leaky integrate-and-fire neuron model in our simulations. The model is defined by a resting membrane potential *V*_*REST*_ and membrane time constant $\tau_{m}$. If the membrane potential surpasses the set threshold potential $\theta_{m}$, it fires a spike and its membrane potential is reset back to *V*_*REST*_. It then enters the refractory period *t*_*ref*_, during which it cannot be stimulated.

The sub-threshold membrane voltage *V*_*i*_ of neuron *i* follows:

$$\tau_{m}\frac{dV_{i}}{dt}=\left(V_{REST}-V_{i}\right)+R\left(g_{ij}^{E}\left(V^{E}-V_{i}\right)+g_{ij}^{I}\left(V^{I}-V_{i}\right)+I_{ex}\right)$$

where *R* is the membrane resistance, $g_{ij}^{E/I}$ are the excitatory and inhibitory synaptic conductances to neuron *i* from neuron *j*, $V^{E/I}$ are excitatory and inhibitory reversal potentials and *I*_*ex*_ is any other externally supplied current. If neuron *i* receives input from neuron *j*, the corresponding synaptic conductance *g*_*ij*_ is as follows:

$$\tau_{E/I}\frac{dg_{ij}}{dt}=-g_{ij}+\bar{g}W_{ij}S_{j}\left(t\right)$$

where $\tau_{E/I}$ is the synaptic time constant, $W_{ij}$ is the synaptic weight, $\bar{g}$ is a basic unit of synaptic conductance and $S_{j}\left(t\right)$ is the presynaptic spike train.

#### Input-dependent inhibitory plasticity (IDIP)

The inhibitory plasticity depends on the synaptic input $y_{i}$ received by each inhibitory neuron i over time.

$$\tau_{IDIP}\frac{dy_{i}}{dt}=-y_{i}+\underset{j}{\sum }g_{ij}^{E}\left(t\right)$$

where $\tau_{IDIP}$ is the input time constant and $g_{ij}^{E}$ is the excitatory synaptic conductance of the synapse from an excitatory neuron j to an inhibitory neuron i. Hence the second term is the sum of all of the excitatory input to the inhibitory neuron i at time t. When the inhibitory neuron i spikes, all the inhibitory synapses projecting from the presynaptic inhibitory neuron i to the postsynaptic excitatory neuron j are adjusted with respect to $y_{i}$ as:

$$\Delta w_{ji}=\eta_{IDIP}\left(y_{i}\left(t\right)-\theta_{in}\right)S_{i}(t)$$

where $\eta_{IDIP}$ is the inhibitory learning rate,$\theta_{in}$ is the constant target input for each inhibitory neuron and $S_{i}(t)$ is the inhibitory neuron i spike train. In our simulations, $\theta_{in}$ is the same for all inhibitory neurons.

#### Different learning rule implementations

In the first variant (’v1’) of the learning rule, we add a voltage term.

$$\tau_{IDIP}\frac{dy_{i}}{dt}=-y_{i}+\sum_{j}g_{ij}^{E}\left(t\right)\left(V^{E}-V_{i}\right)$$

where $V_{i}$ is the sub-threshold membrane voltage of the inhibitory neuron i and $V^{E}$ is the excitatory reversal potential.

In the second variant (’v2′) of the learning rule, we replace the spike train $S_{i}\left(t\right)$ term by a synaptic trace $x_{i}(t)$.

$$\tau_{st}\frac{dx_{i}}{dt}=-x_{i}+S_{i}\left(t\right)$$

$$\Delta w_{ji}=\eta_{IDIP}\left(y_{i}\left(t\right)-\theta_{in}\right)x_{i}\left(t\right)$$

where $\tau_{st}$ is the synaptic trace time constant.

In the third variant (’v3′) of the learning rule, we add inhibitory synapses to the inhibitory neurons in our network and include their synaptic input contribution.

$$\tau_{IDIP}\frac{dy_{i}}{dt}=-y_{i}+\underset{j}{\sum }g_{ij}^{E}\left(t\right)-\underset{j}{\sum }g_{ij}^{I}\left(t\right)$$

where $g_{ij}^{I}$ are the synaptic conductances of the inhibitory synapses to the inhibitory neuron i.

In the fourth variant (’v4’) of the learning rule, we replace the inhibitory synaptic plasticity by plasticity in the inhibitory neuron firing threshold $\theta_{i}$.

$$\Delta \theta_{i}=\eta_{IDIP}\left(y_{i}\left(t\right)-\theta_{in}\right)S_{i}(t)$$

In the fifth variant (’v5′) of the learning rule, we replace the inhibitory neuron target input $\theta_{in}$ with the inhibitory neuron target firing rate $\theta_{x}$. We define a running firing rate estimator $x_{i}^{est}$.

$$\frac{dx_{i}^{est}}{dt}=-\frac{x_{i}^{est}}{\tau_{est}}+S_{i}\left(t\right)$$

where $\tau_{est}$ is the integration time constant, as done in (Wu et al., 2020). The inhibitory weights are updated as:

$$\Delta w_{ji}=\eta_{IDIP}\left(\frac{x_{i}^{est}\left(t\right)}{\tau_{est}}-\theta_{x}\right)S_{i}\left(t\right)$$

The parameters used for each learning rule variant can be found in Tables S1 and S2.

**Table:** 

Neuron Model		
$V_{REST}$	- 60 mV	Resting membrane potential
$\Theta_{m}$	- 50 mV	Membrane potential spiking threshold
R	100 MΩ	Membrane resistance
$t_{ref}$	2 ms	Absolute refractory period
$\tau_{m}$	20 ms	Membrane time constant
$I_{ex}$	0 A	Default external current

Synapse Model		
$\bar{g}$	1 nS	Basic weight unit
$V^{E}$	0 mV	Excitatory reversal potential
$V^{I}$	- 80 mV	Inhibitory reversal potential
$\tau_{E}$	5 ms	Decay constant of AMPA-type conductance
$\tau_{I}$	10 ms	Decay constant of GABA-type conductance
IDIP		
$\tau_{IDIP}$	160 ms	IDIP decay constant

Simulation		
Δt	1 ms	Integration time step size

#### Hippocampal Network Model

We simulate a CA1 network consisting of $N_{E}$ excitatory neurons and $N_{I}$ inhibitory neurons. There is full and bidirectional connectivity between the excitatory and inhibitory neurons. The initial values of the inhibitory synaptic weights ($W_{EI}$ = 1e-3) are set to lower values than that of the excitatory synaptic weights ($W_{IE}$ = 2.0). The CA1 excitatory neurons are divided in $N_{g}$ equally sized groups. Each excitatory neuron in the CA1 network is connected to $N_{CA3}$ CA3 neurons. The excitatory synaptic weights from the CA3 neurons to the CA1 excitatory neurons are made plastic with classical Hebbian plasticity and the inhibitory synaptic weights from the CA1 inhibitory neurons are made plastic with IDIP.

We model a mouse traversing a 1D annular track with $N_{pc}$ equally spaced place fields. We assume the mouse moves with a constant speed and takes 3 s to move from the center of one place field to the center of the subsequent place field.

Each CA3 neuron receives input from one unique place field in the form of an external current $I_{ex}$. The tuning curve T with place field centered at $p_{0}$ is defined as:

$$T\left(p\right)=exp\left(-\frac{{\left(p-p_{0}\right)}^{2}}{2\sigma_{pc}^{2}}\right)$$

where p is the animal’s position and $\sigma_{pc}$ is the tuning width.

The place field current $I_{ex}$ supplied to a CA3 neuron is then:

$$I_{ex}\left(p\right)=A_{c}T\left(p\right)$$

where $A_{c}$ is the current amplitude.

Each CA1 group is tuned to a single unique CA3 neuron, with the same tuning curve T. Within group, the tuning amplitude for each neuron is varied. To this end, we sample a vector of $\frac{N^{E}}{N_{g}}$ random number s from a normal distribution with μ=0 and σ=0.05. We then shuffle this vector for each group and add a single value to the synaptic tuning curve of each neuron in the group.

The excitatory and inhibitory CA1 neurons also receive uniform external currents, $I_{ex}^{E}$ and $I_{ex}^{I}$ respectively.

The excitatory synaptic weights between CA3 and CA1 excitatory neurons follow classical Hebbian plasticity implemented using a symmetrical spike-timing dependent learning rule. A synaptic trace $x_{i}$ is assigned to each neuron i and follows:

$$\tau_{hebb}\frac{dx_{i}}{dt}=-x_{i}+S_{i}(t)$$

where $\tau_{hebb}$ is the time constant of the learning window and $S_{i}t)$ is the spike train. We also include a homeostatic term which takes into account the sum of all synaptic weights onto the postsynaptic neuron. The synaptic weight $w_{ji}$ from the presynaptic neuron i to the postsynaptic neuron j is updated following:

$$\frac{dw_{ji}}{dt}=\eta_{hebb}\left(W_{max}-w_{ji}\right)x_{i}x_{j}-\eta_{homeo}\left(\underset{i}{\sum }w_{ji}-\theta_{homeo}\right)$$

where $W_{max}$ is the maximum excitatory synaptic weight, $\eta_{hebb}$ is the learning rate of the Hebbian term, $\eta_{homeo}$ is the learning rate of the homeostatic term and $\theta_{homeo}$ is the homeostatic target.

**Table:** 

Hippocampal Network Model			
CA3	$N_{CA3}$	10	Size of the CA3 excitatory population
CA1	$N_{E}$	100	Size of the CA1 excitatory population
	$N_{I}$	20	Size of the CA1 inhibitory population
	$N_{g}$	10	Number of excitatory neuron groups
Position modulated input	$N_{pc}$	10	Number of place fields
	$\sigma_{pc}$	6.54 a.u.	Tuning width
	$A_{c}$	200 pA	Current amplitude
External current	$I_{ex}^{E}$	0.01 pA	Current to the excitatory neurons
	$I_{ex}^{I}$	0.5 pA	Current to the inhibitory neurons
Synaptic weights	$W_{IE}$	2.0	E to I synaptic weight
	$W_{EI}$	1e-3	Initial I to E synaptic weight
IDIP	$\eta_{IDIP}$	1e-5 $ms^{-1}nS^{-1}$	IDIP learning rate
	$\theta_{in}$	200 nS	Inhibitory neuron target input
Excitatory plasticity	$\tau_{hebb}$	20 ms	Learning window time constant
	$W_{max}$	1.5	Maximum E to E synaptic weight
	$\eta_{hebb}$	1e-3 $ms^{-1}$	Excitatory Hebbian term learning rate
	$\eta_{homeo}$	1e-4 $ms^{-1}$	Excitatory homeostatic term learning rate
	$\theta_{homeo}$	5.2	Homeostatic target sum of weights

#### Recurrent Network Model

We simulate a recurrent network consisting of $N_{E}$ excitatory and $N_{I}$ inhibitory neurons. The excitatory neurons are randomly connected with a probability $p_{EE}=0.1$, in line with experiments (Harris and Shepherd, 2015). The inhibitory neurons are randomly connected to the excitatory neurons with a probability $p_{IE}=p_{EI}=0.25$. Due to the small size of the network, the *in* degree for each excitatory and inhibitory neuron is kept the same. Thus each excitatory neuron has $N_{E}\times p_{EE}$ excitatory inputs and $N_{I}\times p_{EI}$ inhibitory synaptic inputs. Furthermore, each inhibitory neuron receives $N_{E}\times p_{IE}$ excitatory inputs. Due to the small network size, inhibitory-inhibitory neuron connections are omitted $\left(p_{II}=0\right)$. The values for the recurrent weights $W_{rec}$ are sampled from a log normal distribution, in line with experiments (Loewenstein et al., 2011, Song et al., 2005), with μ=1.0 and $\sigma_{wrec}=0.1$.

The initial values of the inhibitory synaptic weights are ten times weaker than the excitatory weights $\left(W_{I}/W_{E}=0.1\right)$. Furthermore, the inhibitory synaptic weight update $\Delta w_{ji}$ has a multiplicative weight dependence, such that:

$$\begin{array}{cc} w_{ji}\leftarrow w_{ji}+\left(W_{max}-w_{ji}\right)\times \Delta w_{ji} & when\;\Delta w_{ji}>0\\ w_{ji}\leftarrow w_{ji}+w_{ji}\times \Delta w_{ji} & when\;\Delta w_{ji}<0 \end{array}$$

where $W_{max}$ is the is the maximum inhibitory synaptic weight. The inhibitory learning is turned off during the first 15 s of the simulation.

##### Random neuron input generation

Each neuron in the network is randomly connected with a probability of $p_{ex}=0.2$ to a subset of the $N_{ex}$ external inputs, which are modeled as Poisson process with a mean firing rate F. The values of the input synaptic weights from the external inputs to the excitatory and inhibitory neurons $W_{in}$ are sampled from the same log normal distribution as $W_{rec}$, with μ=1.0 and $\sigma_{win}=0.1$. To generate sufficient network activity, we increase the $W_{in}$ by a factor of 2.5. For the simulation in Figure 4B, the values of the select $W_{in}$ are increased by additional 50%.

##### Firing rate rank computation

The same network as above is used in the simulation. We split the activity of the network into 15 s time bins. The first time bin is the network activity before any inhibitory plasticity. We then compute the Spearman’s rank correlation coefficient of the mean excitatory firing rates in the first time bin and each subsequent time bin.

##### Memory protocol

The same network as above is used in the simulation. After the network activity stabilizes (600 s), the recurrent synapses between a selected subset of $N^{E}$ (12/80) are increased by a factor of 5. After the network reaches steady state again (1200 s), the values of the synaptic weights from the $N_{ex}$ to two neurons in the assembly are increased by 50%.

##### Inhibitory spike-timing dependent plasticity (iSTDP)

With iSTDP (Vogels et al., 2011), the synaptic weight $w_{ji}$ from presynaptic inhibitory neuron i and the postsynaptic excitatory neuron j follows:

$$\begin{array}{cc} w_{ji}\leftarrow w_{ji}+\eta_{iSTDP}\left(x_{j}-\alpha \right) & when\;presynaptic\;neuron\;i\;spikes\\ w_{ji}\leftarrow w_{ji}+\eta_{iSTDP}x_{i} & when\;postsynaptic\;neuron\;j\;spikes \end{array}$$

where $\eta_{iSTDP}$ is the learning rate, α is the depression factor and $x_{i/j}$ is the neuron synaptic trace, defined as:

$$\tau_{iSTDP}\frac{dx_{i/j}}{dt}=-x_{i/j}+S_{i/j}(t)$$

where $\tau_{iSTDP}$ is the learning window time constant and $S_{i/j}(t)$ is the spike train.

**Table:** 

Recurrent Network Model			
Neurons	$N_{ex}$	100	Size of the external input population
	$N_{E}$	80	Size of the excitatory population
	$N_{I}$	20	Size of the inhibitory population
Connection probability	$p_{ex}$	0.2	External input to the recurrent
	$p_{EE}$	0.1	Excitatory to excitatory connectivity
	$p_{IE}$	0.25	Excitatory to inhibitory connectivity
	$p_{EI}$	0.25	Inhibitory to excitatory connectivity
	$p_{II}$	0	Inhibitory to inhibitory connectivity
External input	F	10 Hz	Mean firing rate of external Poisson input
Synaptic weights	μ	1.0	Mean of the sampled distribution
	$\sigma_{win}$	0.1	s.d. of the $W_{in}$ sampling distribution
	$\sigma_{wrec}$	0.1	s.d. of the $W_{rec}$ sampling distribution
	$W_{I}/W_{E}$	0.1	Initial inhibitory/ excitatory weight ratio
IDIP	$\eta_{IDIP}$	1e-7 $ms^{-1}nS^{-1}$	IDIP learning rate
	$\theta_{in}$	550 nS	Inhibitory neuron target input
	$W_{max}$	1.0	Maximum I to E synaptic weight
iSTDP	$\tau_{iSTDP}$	20 ms	Learning window time constant
	$\eta_{iSDTP}$	5e-5 $ms^{-1}$	iSTDP learning rate
	α	0.2	Depression factor

#### Sparse Inhibitory Connectivity Network Model

We simulate a network of 2 excitatory and 2 inhibitory spiking neurons. The neurons are arranged on a ring, with the excitatory neurons providing di-synaptic inhibition to each other (Figure S3J). Each neuron receives additional external current. One excitatory neuron receives slightly (5%) stronger external current. The values of all the parameters used can be found in Table S3.

### Quantification and statistical analysis

#### Quantification

All network simulations (apart from Figures 4G and 4H) were performed with 20 different network connectivities and/or input random seeds.

#### Statistical analysis

Spearman rank (Figure 4E) and Pearson (Figure S4B) correlation coefficient was used to quantify the maintenance of neural representation after inhibitory learning. A Student’s t test was used to quantify the difference between different learning rule variants in recurrent networks (Figure S4C).

#### Software

All software used (Python & Numpy) is freely available. The specific versions used are listed in the Key resources table.

## Data Availability

• This study did not generate data or analyze existing datasets. • All original code has been deposited at https://github.com/klarakaleb/IDIP and is publicly available as of the date of publication. • Any additional information required to reanalyze the data reported in this paper is available from the lead contact upon request.

## Supplemental information

Document S1. Figures S1–S4 and Tables S1–S3

Document S2. Article plus supplemental information
