# Supplementary material for: Network-centered homeostasis through inhibition maintains hippocampal spatial map and cortical circuit function
Source: Cell Rep. 2021 Aug 24;36(8):109577. doi: 10.1016/j.celrep.2021.109577 (PMC8411119; doi:10.1016/j.celrep.2021.109577)
Supplement: Document S1. Figures S1–S4 and Tables S1–S3 [file mmc1.pdf]

Cell Reports, Volume *36*

Supplemental information

Network-centered homeostasis through inhibition maintains hippocampal spatial map and cortical circuit function

Klara Kaleb, Victor Pedrosa, and Claudia Clopath

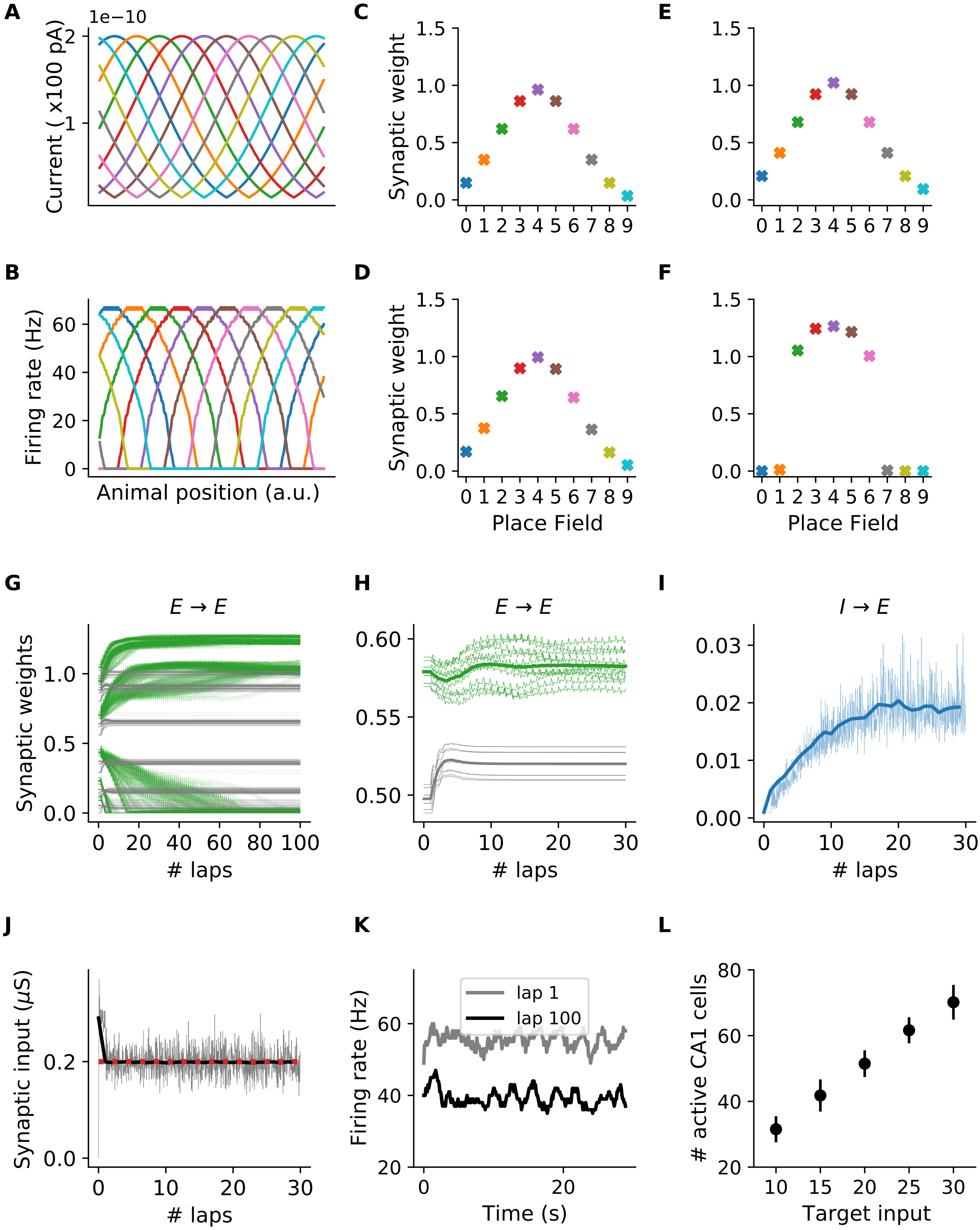

A
1e−10
Current (x100 pA)
B
Firing rate (Hz)
Animal position (a.u.)
C
Synaptic weight
D
Synaptic weight
Place Field
E
F
Place Field
G
E → E
Synaptic weights
laps
H
E → E
laps
I
I → E
laps
J
Synaptic input (μS)
laps
K
Firing rate (Hz)
lap 1
lap 100
Time (s)
L
active CA1 cells
Target input

Figure S1. The IDIP rule enables emergence of active and silent place cells in a hippocampal network. Related to Figure 1. A The external current supplied to the CA3 neurons as a function of simulated animal position. Different colours indicate different place fields. **B** The CA3 neuron firing rates as a function of simulated animal position. Different colours indicate different CA3 neurons tuned to different place fields, as in **A**. **C** The initial CA3 to CA1 synaptic weights of cell 1, as in Fig 1.C. Different colours indicate synapses from CA3 neurons with different place fields, as in S1. **D** The final CA3 to CA1 synaptic weights of neuron 1, as in Figure 1.F. **E-F** Same as in **C-D** but for cell 2. **G** CA3 to CA1 synaptic weights of all active (green) and silent (gray) place cells as a function of time. **H** Mean CA3 to CA1 synaptic weights of an all active (green) and silent (gray) place cells as a function of time. The bold lines denote the mean over all active (silent) cells per lap. **I** CA1 inhibitory synaptic weights as a function of time. The bold lines denote the mean value per lap. **J** The synaptic input to the inhibitory neurons (gray line) as a function of time. The bold black line denotes the mean value per lap. The red dotted line denotes the target input. **K** Firing rate of the CA1 inhibitory neurons in the first (gray) and last (black) lap of the simulation. **L** The mean number of active place cells as a function of target input value. The error bars correspond to $\pm$ STD over 20 networks.

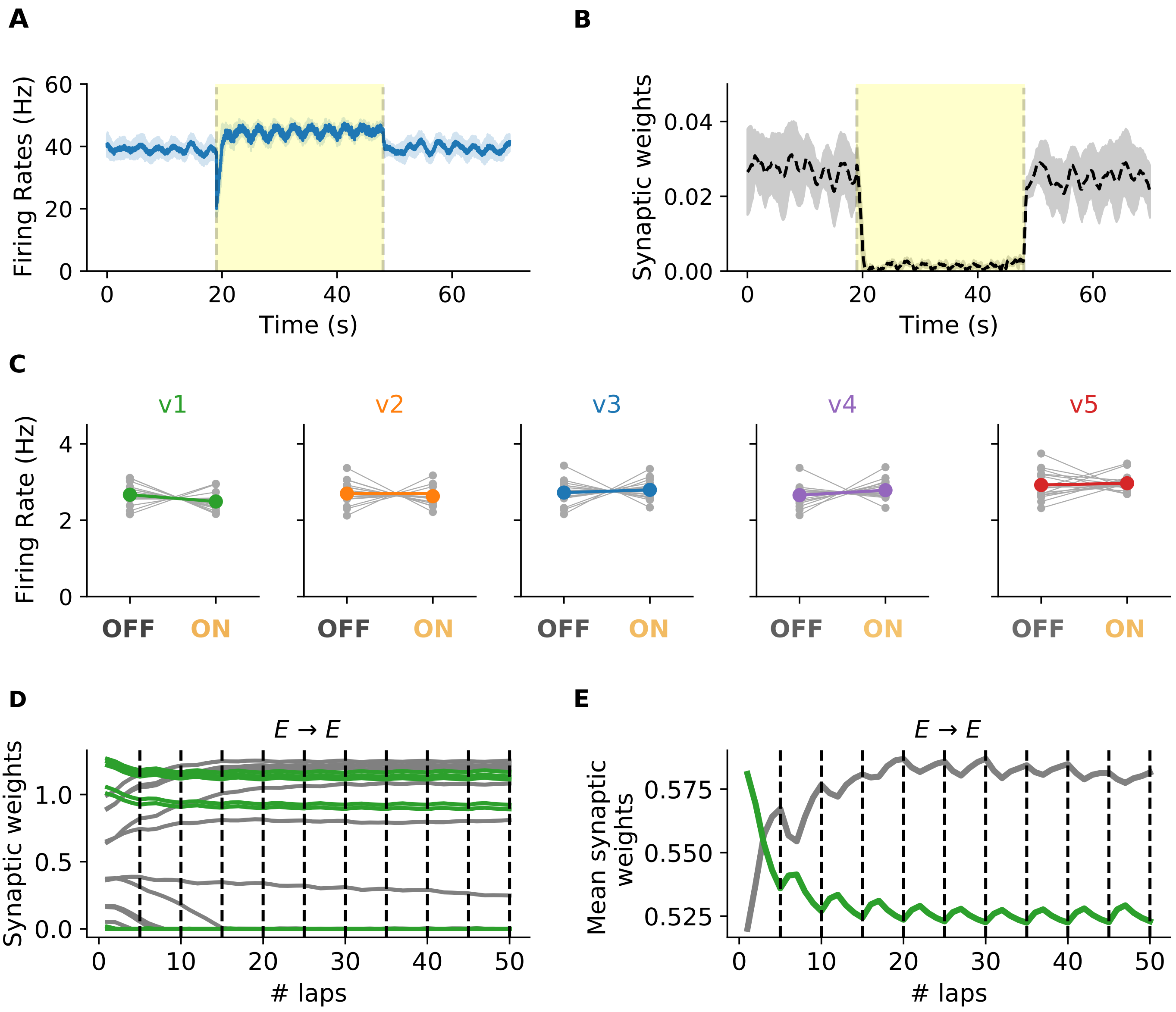
A
Firing Rates (Hz)
Time (s)
B
Synaptic weights
Time (s)
C
v1
v2
v3
v4
v5
Firing Rate (Hz)
OFF
ON
D
E → E
Synaptic weights
laps
E
E → E
Mean synaptic weights
laps

Figure S2. Place cells silencing leads to rapid emergence of an alternative place map. Related to Figure 2. A Evolution of the mean inhibitory firing rates during the silencing protocol as described in Figure 2A Shaded area corresponds to $\pm$ STD across 20 networks. **B** Evolution of the mean inhibitory synaptic weights during the silencing protocol as described in Figure 2A. Shaded area corresponds to $\pm$ STD across 20 networks. **C** Same as in Figure 2E but for the networks following IDIP learning rule variants. Gray circles indicate individual networks. Colour-coded circles indicate average over 20 networks. **D** The evolution of all the synaptic weights from CA3 to CA1 for an example place (green) and silent (gray) cell during the consolidation protocol as described in Figure 2I. Dashed vertical lines mark testing laps. **E** Same as in **D** , but for the mean synaptic weights an example place (green) and silent (gray) cell.

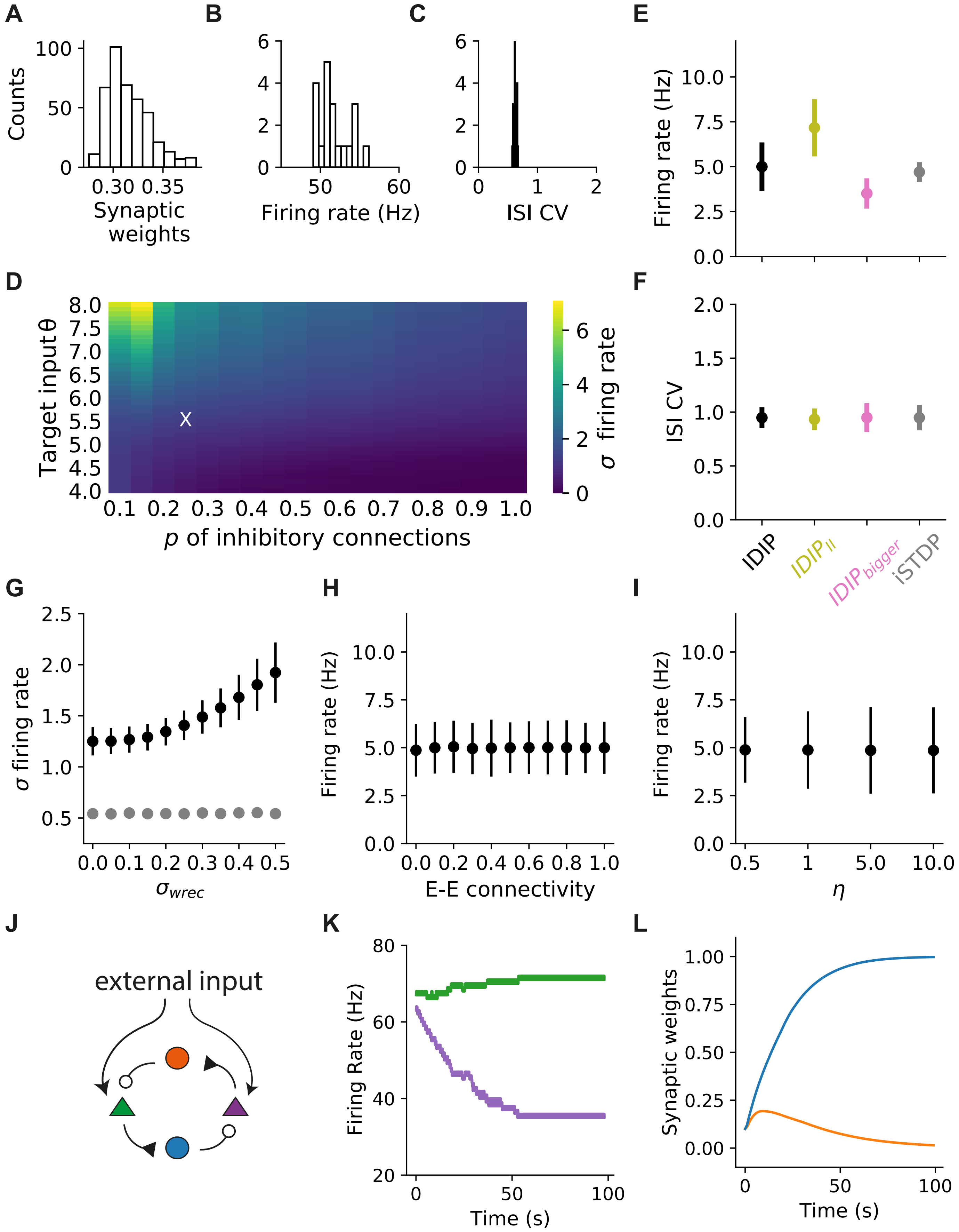

A
Counts
Synaptic weights
B
Firing rate (Hz)
C
ISI CV
E
Firing rate (Hz)
D
Target input θ
p of inhibitory connections
σ firing rate
X
F
ISI CV
IDIP
IDIP II
IDIP bigger
iSTDP
G
σ firing rate
σwrec
H
Firing rate (Hz)
E-E connectivity
I
Firing rate (Hz)
η
J
external input
K
Firing Rate (Hz)
Time (s)
L
Synaptic weights
Time (s)

Figure S3. IDIP establishes global E/I balance in recurrent networks. Related to Figure 3. **A** The final inhibitory synaptic weights distribution. **B** The final inhibitory firing rate distribution. **C** The final inhibitory ISI CV distribution. **D** The standard deviation of the firing rates after IDIP as a function of different values of inhibitory probability of connections (x axis) and the target input θ_i (y axis). 'X' marks the parameter combination used in our simulations. **E** The mean firing rate after inhibitory learning for the original network (IDIP), a network with fixed inhibitory-inhibitory connections ($IDIP_{II}$), a bigger network (500 neurons, $IDIP_{bigger}$) and a network following iSTDP. Data collated over 20 networks. Error bars correspond to ± STD. **F** Same as in **E** but for ISI CV. **G** Standard deviation for the networks following IDIP (black) and iSTDP (gray) as a function of the standard deviation of the W_{rec} sampling distribution (σ_{wrec}). Error bars correspond to ± STD. **H** Mean firing rate of the networks following IDIP as a function of excitatory to excitatory (E-E) connectivity. **I** Mean firing rate of the networks following IDIP as a function of inhibitory learning rate η. **J** Diagram of a network with sparse inhibitory connectivity. Two excitatory (triangles) and two inhibitory (circles) neurons are simulated. One of the excitatory neurons (green) receives a slightly (5%) stronger external input. Thus one inhibitory neuron (blue) receives synaptic input from the excitatory neuron that is more active and projects onto the other excitatory neuron (purple). The other inhibitory neuron (orange) receives synaptic input from the less active excitatory neuron (purple) and projects onto the more active (green) excitatory neuron. **K** The evolution of neuronal firing rates for the excitatory cells, color coded as in **J**. The initially small activity difference is amplified by the inhibitory plasticity, leading to increased activity of one excitatory neuron (green) and a decrease in the other (purple). **L** The evolution of the inhibitory (I to E) synaptic weights, color coded as in **J**.

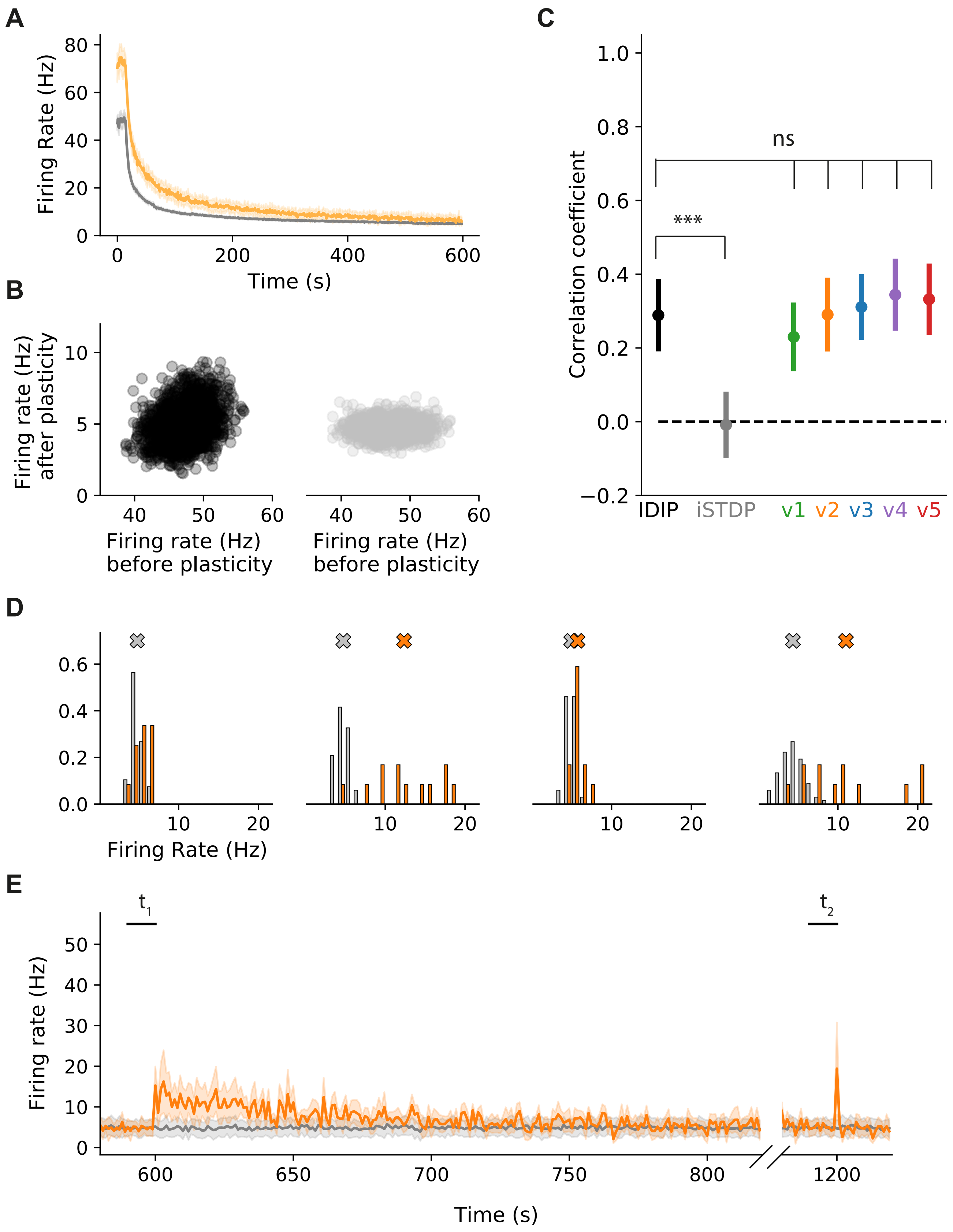

A
Firing Rate (Hz)
Time (s)
B
Firing rate (Hz) after plasticity
Firing rate (Hz) before plasticity
Firing rate (Hz) before plasticity
C
Correlation coefficient
ns
***
IDIP
iSTDP
v1
v2
v3
v4
v5
D
Firing Rate (Hz)
E
t_1
t_2
Firing rate (Hz)
Time (s)

Figure S4. IDIP rule enables maintenance of neural representations and memory trace persistence. Related to Figure 4. **A** Same as Figure 4B but in a network following iSTDP. **B** Recurrent network as in Figure 3A, with no neurons receiving increased external input. The excitatory firing rate before (x-axis) versus after (y-axis) inhibitory learning in the network following IDIP/iSTDP (black/gray) (Pearson correlation coefficient $r = 0.29$, p-value = 5.09e-32 (IDIP); $r = 0.005$, p-value = 0.84 (iSTDP)). **C** The final firing rate rank correlation coefficient, calculated as in Figure 4E, for the recurrent networks following different IDIP learning rule variants. The correlation coefficient values of the networks following IDIP and iSTDP are significantly different (student t-test, p value = 7e-12). The correlation coefficient values of the networks following IDIP and IDIP learning rule variants are not significantly different (student t-test, p values = 0.07 (v1), 0.96 (v2), 0.47 (v3), 0.09 (v4), 0.18 (v5)). The error bars correspond to $\pm$ STD over 20 networks. **C-D** Same as Figure 4G-H but in a network following iSTDP.

Supplementary table 1. The parameters for the different learning rule variants in the hippocampal networks, as described in STAR methods.

Hippocampal network			
**v1**	η_{IDIP}	4e-4 $ms^{-1}nS^{-1}$	IDIP learning rate
	θ_{in}	-1.0 nA	Inhibitory neuron target input
**v2**	η_{IDIP}	5e-7 $ms^{-1}nS^{-1}$	IDIP learning rate
	τ_{st}	20 ms	Synaptic trace time constant
**v4**	η_{IDIP}	5e-6 $ms^{-1}mV^{-1}$	IDIP learning rate
	W_{EI}	1e-2	Initial I to E synaptic weight
**v5**	η_{IDIP}	1e-4 $ms^{-1}\text{Hz}^{-1}$	IDIP learning rate
	τ_{est}	20 ms	Firing rate estimator time constant
	θ_x	80 Hz	Inhibitory neuron target firing rate

Supplementary table 2. The parameters for the different learning rule variants in the recurrent networks, as described in STAR methods.

Recurrent network			
**v1**	η_{IDIP}	4e-6 $ms^{-1}nS^{-1}$	IDIP learning rate
	θ_{in}	-3.0 nA	Inhibitory neuron target input
**v2**	η_{IDIP}	5e-9 $ms^{-1}nS^{-1}$	IDIP learning rate
	τ_{st}	20 ms	Synaptic trace time constant
**v4**	η_{IDIP}	5e-10 $ms^{-1}mV^{-1}$	IDIP learning rate
	W_I/W_E	0.15	Inhibitory/excitatory weight ratio
**v5**	η_{IDIP}	1e-6 $ms^{-1}\text{Hz}^{-1}$	IDIP learning rate
	τ_{est}	20 ms	Firing rate estimator time constant
	θ_x	100 Hz	Inhibitory neuron target firing rate

Supplementary table 3. The parameters for the network with sparse inhibitory connectivity, as described in STAR methods.

Recurrent Network Model			
**Neurons**	N_E	2	Size of the excitatory population
	N_I	2	Size of the inhibitory population
**Ex. input**	I_E	200/210 pA	External current to the excitatory neurons
	I_I	20 pA	External current to the inhibitory neurons
**Synaptic weights**	W_{IE}	7.0	E to I synaptic weight
	W_{EI}	0.1	Initial I to E synaptic weight
**IDIP**	η_{IDIP}	1.5e-5 $ms^{-1}nS^{-1}$	IDIP learning rate
	θ_{in}	350 nS	Inhibitory neuron target input
